# The role of sex, adiposity, and gonadectomy in the regulation of irisin secretion

**DOI:** 10.1007/s12020-016-0913-x

**Published:** 2016-04-07

**Authors:** M. Zügel, S. Qiu, R. Laszlo, E. Bosnyák, C. Weigt, D. Müller, P. Diel, J. M. Steinacker, U. Schumann

**Affiliations:** 1 Division of Sports Medicine, Department of Internal Medicine, University Hospital Ulm, Parkstr. 11, 89075 Ulm, Germany; 2 Department of Endocrinology, Zhongda Hospital, School of Medicine, Southeast University, Nanjing, People’s Republic of China; 3 Department of Health Sciences and Sports Medicine, University of Physical Education, Budapest, Hungary; 4 Department of Molecular and Cellular Sports Medicine, German Sports University Cologne, Cologne, Germany

**Keywords:** Irisin, PGC-1α, FNDC5, Skeletal muscle, Ovariectomy, Orchiectomy, Obesity, Sex hormones

## Abstract

A sexual dimorphism has been reported for the adipo-myokine irisin at rest and in response to exercise. The effects of male and female sex, adiposity, and gonadectomy on irisin secretion have not been investigated before. The objective of this study was to elucidate the effects of sex, adiposity, and gonadectomy in the regulation of irisin secretion as well as PGC-1α/FNDC5 mRNA and protein expression. We hypothesized that a lack of female sex hormones by ovariectomy reduces irisin levels and inhibits skeletal muscle expression of PGC-1α and FNDC5. Circulating irisin was measured in vivo in serum samples of healthy and obese men and women at rest and in response to acute exercise. The effects of gonadectomy on serum irisin, PGC-1α and FNDC5 muscle mRNA, and protein expression were investigated in ovariectomized (OVX) and orchiectomized (ORX) Wistar rats. Serum irisin at rest was not significantly different between men and women (lean or obese). However, in response to acute aerobic exercise, irisin levels increased significantly more in lean women versus men (*p* ≤ 0.05). In obese individuals, resting irisin concentrations were significantly higher compared to lean subjects (*p* ≤ 0.001) and the irisin response to acute exercise was blunted. Only the lack of gonadal hormones in OVX but not ORX rats increased serum irisin levels (*p* ≤ 0.01) and resulted in significantly increased body weight (*p* ≤ 0.01), adipose tissue content (*p* ≤ 0.05), muscle FNDC5 mRNA (*p* ≤ 0.05), and protein (*p* ≤ 0.01) expression without altering PGC-1α expression. Testosterone treatment in ORX rats leads to increased PGC-1α mRNA content and reduced PGC-1α protein content without affecting FDNC5 expression or serum irisin levels. We show that a sexual dimorphism exists for the acute irisin response to exercise in normal-weight but not in obese subjects. OVX, which is associated with increased adiposity and insulin insensitivity, increases basal FNDC5 expression and serum irisin, without altering PGC-1α expression. This may be an early sign for metabolic disturbances associated with menopause, such as a developing irisin resistance or an attempt of the organism to improve glucose metabolism.

## Introduction

Maintaining energy homeostasis in response to metabolic perturbations is a major challenge for any multicellular and complex organism involving multiple and sometimes redundant physiological responses [1]. The identification of brown (BAT) and brown-in-white (brite) adipose tissue in humans [2, 3] has sparked major interests in BAT biology, with the long-term goal to develop treatment strategies for metabolic diseases. An elaborate communication network exists between stimuli such as cold exposure, sympathetic activation, and factors secreted from metabolically active organs such as skeletal muscle (myokines) and adipose tissue (adipokines), working together to regulate BAT and brite adipose tissue activity. Adipo-myokines are considered to mediate many of the beneficial effects of regular exercise for human health. Although the precise mechanisms are still under investigation, new studies show that cross-talk between the skeletal muscle and adipose tissue secretomes with various tissues and organ systems may play an important role for metabolic function, body weight regulation, and brain health [4].

The novel adipo-myokine irisin was identified by Bostrom et al. in a search for PGC-1α target genes coding for secreted proteins. They identified the transmembrane protein FNDC5 (fibronectin [type 3] domain-containing [protein] 5) as a PGC-1α target, which is cleaved to produce soluble irisin. They later used adenoviral overexpression of FNDC5 in the liver of mice to show that transgenic mice exhibited enhanced oxygen consumption, increased amounts of white adipose tissue (WAT) browning, and improved glucose tolerance and insulin sensitivity [5]. There may however be other molecules regulating FNDC5/irisin other than PGC-1α, since increased PGC-1α after electrical stimulation of myotubes, exercise mimetics, or exercise training in vivo does not necessarily lead to an activation of FNDC5 expression [6–9]. Recently, the extracellular signal-related kinase (ERK) signaling pathway has been suggested to control FNDC5 expression through a non-genomic pathway during neural differentiation of mouse embryonic stem cells [10]. In turn, the browning effects of irisin have been shown to depend on the activation of ERK and p38 protein kinase signaling cascades [11]. Activation of AMPK may also be involved in FNDC5/irisin activation. FNDC5 expression in skeletal muscle was shown to be dramatically reduced in resting muscles of AMPK muscle-specific knockout mice compared to wild-type mice [12]. In response to muscle contractions, AMPK and FNDC5 activation were abolished even though PGC-1α was increased in wild-type and knockout mice, suggesting that AMPK may be required for the regulation of FNDC5 independent of PGC-1α. Interestingly, myostatin knockout mice exhibit not only increased muscle mass but also browning of WAT, which was shown to be driven by AMPK [13]. Inhibition of AMPK significantly reduced PGC1α and FNDC5 expression [13]. However, other studies do not confirm a critical role for AMPK in the regulation of irisin secretion. Treatment with the anti-diabetic drug metformin increased intramuscular FNDC5 expression, and circulating irisin and AMPK inhibition did not abolish this effect [14]. The regulation and the physiological actions of irisin are not yet fully understood, and further studies are required to identify putative irisin receptors and study the underlying mechanisms responsible for activating FNDC5/irisin.

Irisin is predominantly secreted from skeletal muscle, subcutaneous, and visceral adipose tissue [15, 16]. However, a recent immunohistochemical study showed that a number of other tissues such as the liver, brain, testis, spleen, and the stomach are also able to produce irisin [17].

Numerous studies have investigated the potential factors triggering irisin secretion. Acute aerobic exercise has been shown to transiently increase irisin levels in humans [7, 18–20] and mice [21]. A recent study suggests that resistance exercise induces a greater irisin response than endurance exercise [22]. However, this may be due to the low endurance exercise intensity performed in this study. Exercise intensity appears to be a relevant factor for irisin secretion since no changes in serum irisin levels were detected after low intensity exercise [8, 23]. In contrast to acute exercise, chronic exercise training has either no effects or even lowers resting irisin levels [7]. A recently published meta-analysis from our group revealed that chronic exercise training was associated with a moderate and significant overall effect on decreasing circulating irisin compared with controls [24]. Exercise training chronically stimulates numerous signaling molecules and cascades acting in concert to improve skeletal muscle substrate uptake and oxidation, such as GLUT-4 expression and translocation [25, 26], perhaps reducing the need for elevated resting irisin levels or increasing irisin sensitivity. Interestingly, shivering due to cold exposure also stimulates irisin secretion proportional to shivering intensity and similar in magnitude to intense acute exercise [27]. Also, acute bouts of whole-body vibration exercise transiently elevate circulating irisin levels [28]. Together, these findings suggest that muscle contraction is the main stimulus for irisin secretion.

Irrespective of the controversy regarding a point mutation within the start codon of FNDC5 and its consequences for the secretion of irisin in humans [9], irisin has the potential to be developed as a pharmacological agent, since injections of recombinant irisin in obese, diabetic mice led to significant reductions in body weight, improved glucose homeostasis [11], and reduced triglyceride levels [11].

Recent data advocate the existence of a sexual dimorphism regarding irisin secretion levels at rest, which were shown to be higher in girls versus boys [29], and in women versus men [30]. Furthermore, after 3 weeks of sprint interval training, irisin levels were increased in women and slightly decreased in men [31]. Our hypothesis that a lack of female sex hormones by ovariectomy reduces irisin levels and inhibits skeletal muscle expression of PGC-1α and FNDC5 is supported by findings of a positive association between 17β-estradiol (E2) and irisin [32].

To date, no data exist on the effects of sex on irisin levels in response to acute exercise in lean and obese participants and on the lack of sex hormones by gonadectomy on irisin secretion. Therefore, the aim of this study was to further elucidate the role of sex, adiposity, and gonadectomy on circulating irisin levels and skeletal muscle PGC1α/FNDC5 expression in (I) lean and obese men and women at rest and in response to acute exercise and (II) ovariectomized (OVX) or orchiectomized (ORX) Wistar rats.

## Methods

### Experimental design

#### Human study

Sixteen lean (nine women, 26.3 ± 5.4 years, 65.4 ± 6.5 kg; seven men, 25.8 ± 6.8 years, 86.4 ± 10.2 kg) and 12 morbidly obese participants (seven women, 52.8 ± 14 years, 128.7 ± 37.1 kg; five men, 54.2 ± 8.1 years, 158.8 ± 26.9 kg) were recruited to perform acute endurance exercise protocols. Lean study participants accomplished a step-wise incremental exercise trial until exhaustion on a cycling ergometer (Excalibur Sport, Netherlands). A modified treadmill walking protocol was used for obese participants, which led to exhaustion in approximately 10 min. Venous blood was drawn prior to and immediately post exercise in obese and additionally 10 and 60 min post exercise in lean participants. Capillary blood samples (20 µl) were taken from the hyperemized ear lobe and analyzed for blood lactate (Biosen S-Line, EKF; Barsleben, Germany). Serum samples were obtained by centrifugation for 10 min at 2500×*g* and were stored at −80 °C for subsequent analysis. All experiments involving human participants were conducted with ethical approval from the ethics committee at Ulm University. All study participants gave written informed consent after the study procedures and objectives were explained.

#### Animals and tissue preparation

Male and female Wistar rats (*n* = 6/group) were kept at constant room temperature (20 ± 1 °C), relative humidity (50–80 %), and illumination (12 h light/dark cycles). Water and a soybean free diet (Ssniff^®^ Sm R/M-H, 10 mm, phytoestrogen-free, Soest, Germany) were provided ad libitum. Before the start of experimental procedures, animals were either left intact (intact) or were ovariectomized (OVX), orchiectomized (ORX), or sham operated (sham) via the dorsal route. A subset of ORX rats was treated with testosterone propionate (1 mg/kg BW) for 10 days after 7 days of hormonal decline. Animals were sacrificed by decapitation after light anesthesia with CO_2_ inhalation at the age of 18 weeks (female Wistar rats) or 14 weeks (male Wistar rats). Skeletal muscle (*M. soleus*) and intra-abdominal visceral fat (retroperitoneal and mesenteric fat pads) were dissected, wet weights were recorded, and tissues were directly frozen in liquid nitrogen until further analysis. All animal experimentation described in the manuscript was conducted after ethics approval in accord with accepted standards of humane animal care.

### Analytical methods

#### Irisin measurement

Irisin concentrations in serum were determined using a competitive, commercially available ELISA kit (Phoenix, EK-067-52). The linear range for this assay is 0.066–1024 ng/ml (intra-assay variation 4–6 %, inter-assay variation 8–10 %). Absorbance was measured using a spectrophotometer at 450 nm wavelength (Thermo Scientific, Multiskan FC).

#### qPCR experiments

Total RNA from frozen muscle was extracted from each animal (*n* = 6 animals/group) using the Qiagen Tissue Ruptor in combination with the Qiagen RNeasy Fibrous Tissue Mini Kit. RNA was quantified by spectrophotometry (NanoDrop TM 1000, Thermo Scientific, USA), and cDNA was synthesized from 1 μg RNA using a Reverse Transcription System (QuantiTect^®^ Reverse Transcription, Qiagen). qPCR was performed using the QuantiFast SYBR Green PCR Kit (Qiagen, Germany) on a Lightcycler^®^ 480 (Roche) platform. QuantiTect^®^ Primer Assays (Qiagen) were used to detect *PGC-1α* and *FNDC5* in rat skeletal muscle. Relative expressions of target genes from three independent PCR experiments were calculated after normalization to the endogenous reference gene *RPL13* according to the ΔΔCT method.

#### Protein expression

Proteins were isolated from frozen muscle samples after disruption and homogenization (Qiagen TissueRuptor^®^) followed by sonification (Bandelin, Sonoplus) using RIPA buffer (25 mM Tris–HCl pH 7.6, 150 mM NaCl, 1 % NP-40, 1 % sodium deoxycholate, 0.1 % SDS, Pierce) plus protease inhibitor (cOmplete, Mini, EDTA-free tablets, Roche). Protein concentration was determined by the BCA assay (Pierce). Protein lysates (*n* = 6 animals/group pooled) were subjected to SDS–polyacrylamide gel electrophoresis (PAGE, Bio-Rad) and blotted onto a PVDF membrane (Amersham) for 45 min (300 mA). Membranes were incubated with rabbit anti-PGC-1α (1:1000; Millipore), rabbit FNDC5 (1:500; Abgent), and mouse β-Actin (1:5000; Calbiochem) primary antibodies at 4 °C overnight. After a 1-h incubation period with secondary anti-rabbit (1:3500; Promega) or anti-mouse (1:10,000; Calbiochem) HRP-conjugated antibody, specific proteins were visualized with the ECL detection system (Amersham). Three independent blots were quantified by densitometry (Image J software), and results were expressed as signal intensities relative to the expression of the endogenous reference protein β-Actin.

### Statistical analysis

Statistical significance of differences were calculated using either two-way ANOVA with Tukey’s multiple comparison test to determine the effects of sex and exercise or one-way ANOVA to assess the effects of hormone status between groups (GraphPad Prism, 6.0). Statistical significance was established at *p* ≤ 0.05.

## Results

### Sexual dimorphism in the irisin response to acute endurance exercise in normal-weight but not in obese subjects

**Fig. 1:**
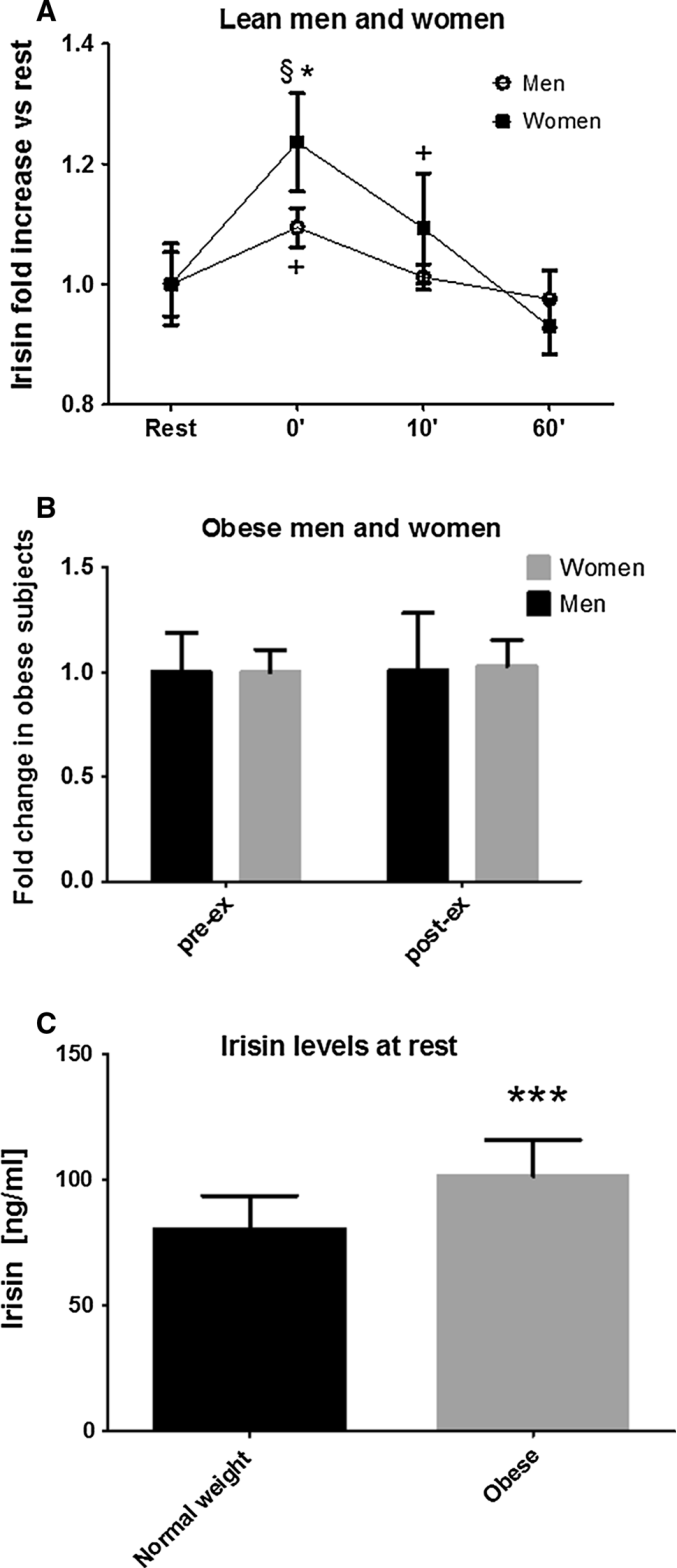
Effects of sex and obesity on resting and exercise-induced irisin secretion. Circulating irisin concentrations increase in normal-weight (**a**) but not in obese men and women (**b**) immediately after acute aerobic exercise. Resting irisin levels are higher in obese versus normal-weight participants (**c**). **a** *p ≤ 0.05 women versus men immediately after exercise, ^§^ *p* ≤ 0.01 women 0′ vs rest, ^+^ *p* ≤ 0.05 men 0′ versus rest and women 10′ versus rest, **c** ***p ≤ 0.001 obese versus normal-weight subjects at rest

Differences in irisin concentrations at rest between men and women were non-significant, neither in lean individuals (74.38 vs. 84.02 ng/ml) nor in obese persons (101.42 ± 19.2 vs. 101.97 ± 11.0 ng/ml). However, irisin levels increased significantly after acute cycling exercise until exhaustion in lean men (*p* ≤ 0.05) and women (*p* ≤ 0.01) (Fig. 1a), while no significant change was observed after exercise in neither obese men (101.4 pre-ex vs. 102.1 ng/ml post-ex) nor women (102.0 pre-ex vs. 105.0 ng/ml post-ex) (Fig. 1b). Elevated irisin levels in lean subjects returned to baseline levels 10′ (men) or 60′ (women) after exercise (Fig. 1a). There was a significant main effect of sex and time (*p* ≤ 0.05) with a greater increase in irisin concentrations and slower return to baseline levels after exercise in lean women versus men. Furthermore, a significant difference in the concentration of irisin between obese versus lean men and women was detected at rest (*p* ≤ 0.001). Resting irisin concentrations were approximately 30 % higher in obese versus lean individuals (Fig. 1c).

### Ovariectomy (OVX) increases body weight, visceral fat content, and serum irisin levels

**Fig. 2:**
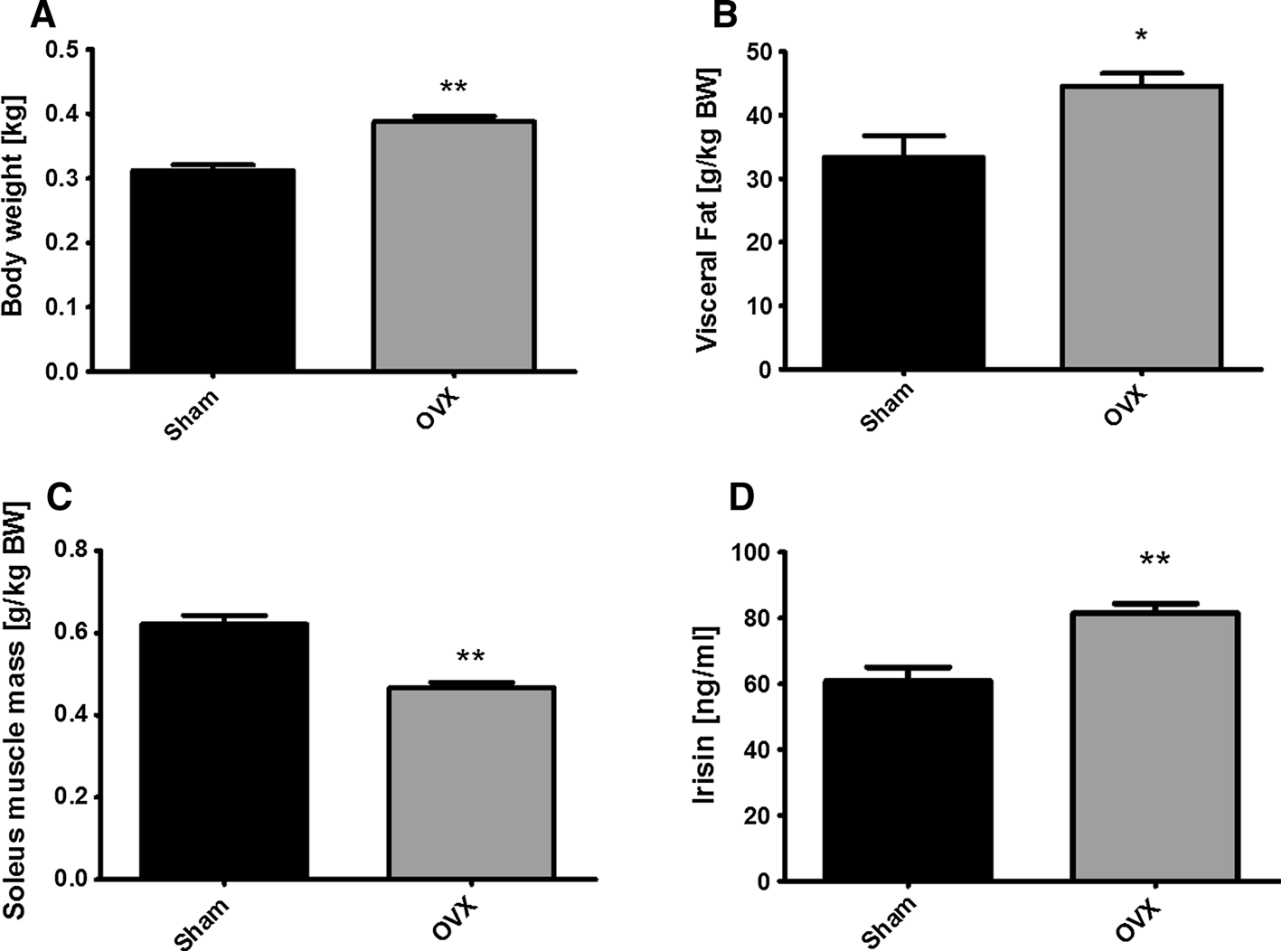
Effects of ovariectomy (OVX) on body weight, visceral fat content, muscle mass and serum irisin levels. Body weight (**a**), visceral body fat (**b**), muscle mass relative to body weight (**c**) and serum irisin levels (**d**) in sham-operated versus ovariectomized (OVX) Wistar rats. *p ≤ 0.05 and **p ≤ 0.01 OVX versus sham

Body weight was significantly higher in OVX versus sham-operated Wistar rats (Fig. 2a, *p* ≤ 0.01). Relative to body weight, visceral fat content (Fig. 2b, *p* ≤ 0.05) was significantly higher and muscle mass (Fig. 2c, *p* ≤ 0.01) significantly lower in OVX versus sham rats. Resting irisin levels were ~25 % higher in female OVX rats compared with sham rats (Fig. 2d, *p* ≤ 0.01).

### Orchiectomy (ORX) does not affect serum irisin levels nor phenotype

**Fig. 3:**
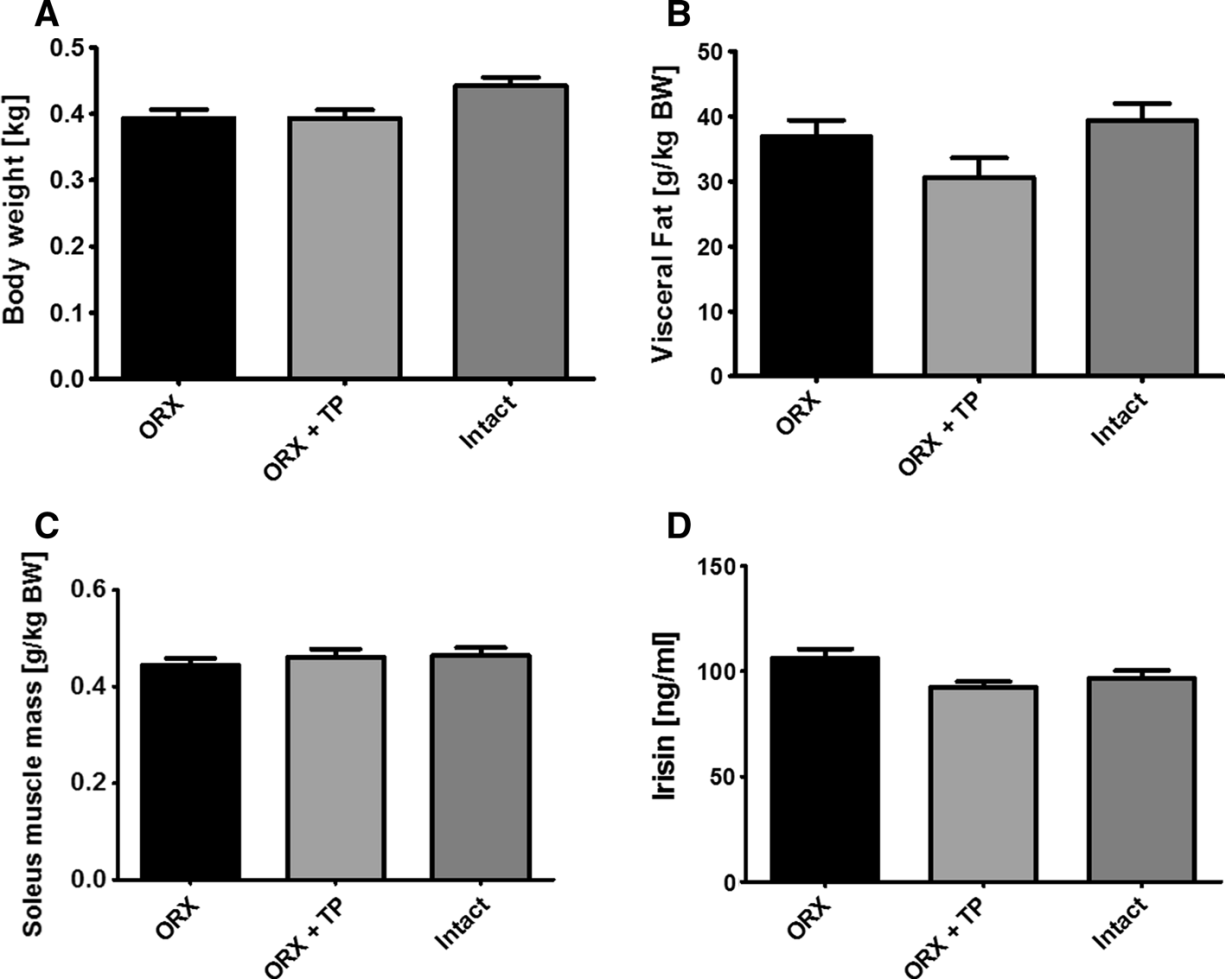
Effects of orchiectomy (ORX) on body weight, visceral fat content, muscle mass, and serum irisin levels. Body weight (**a**), visceral body fat (**b**), muscle mass relative to body weight (**c**), and serum irisin levels (**d**) in orchiectomized (ORX) versus ORX + testosterone propionate (TP) treated and gonad intact (intact) male Wistar rats, *n* = 6/group

ORX in male Wistar rats was not associated with significant changes in body weight, visceral body fat content, skeletal muscle mass, or serum irisin levels compared to gonad intact rats (Fig. 3).

### Ovariectomy (OVX) increases FNDC5 mRNA and protein content

**Fig. 4:**
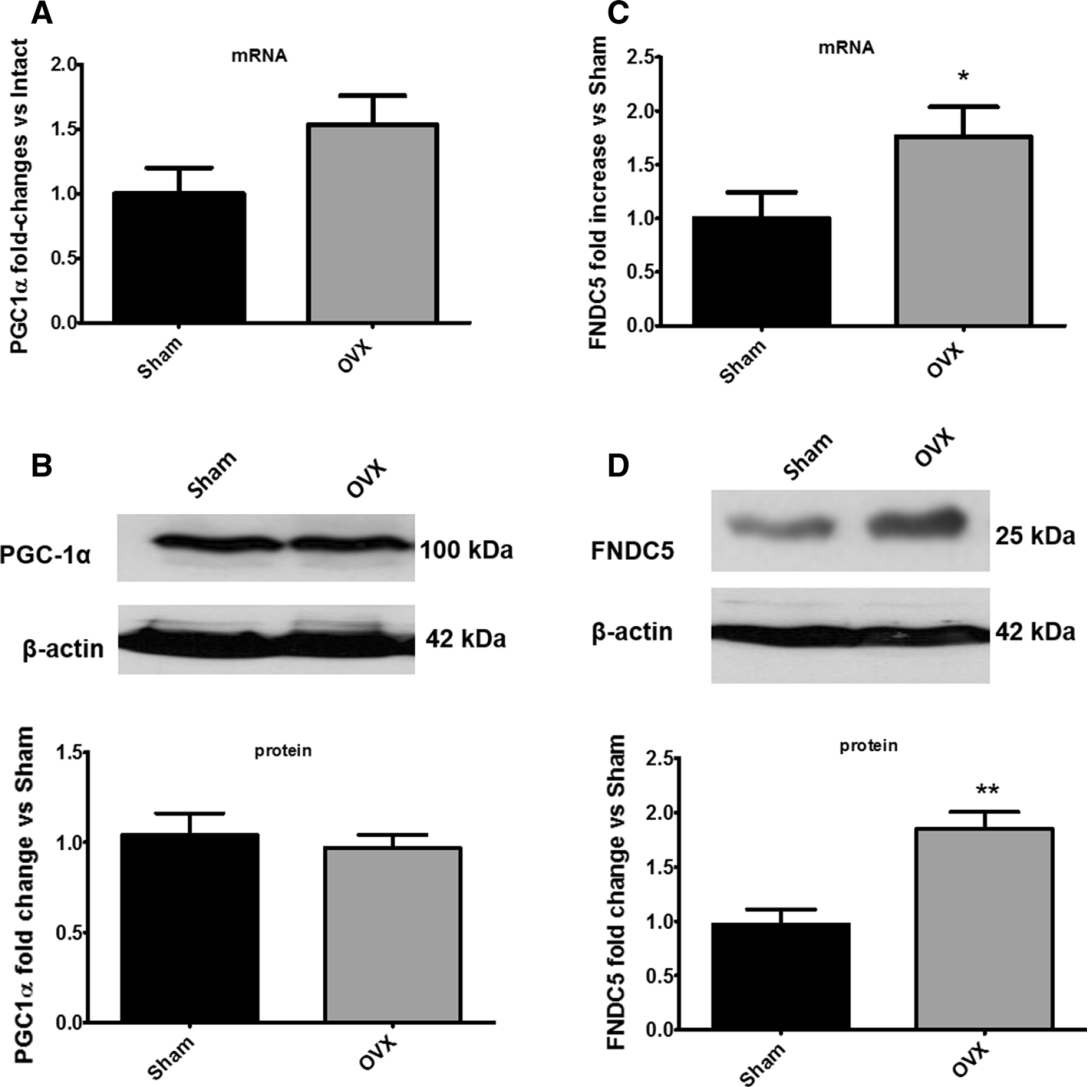
Ovariectomy (OVX) increases FNDC5 mRNA and protein content. mRNA expression of PGC-1α (**a**) and FNDC5 (**c**). Western blot and densitometric analysis of PGC-1α (**b**) and FNDC5 (**d**) in skeletal muscle of sham-operated and ovariectomized (OVX) female Wistar rats, *n* = 6/group. *p ≤ 0.05 OVX versus sham, **p ≤ 0.01 OVX versus sham

Resting PGC-1α mRNA (Fig. 4a) or protein (Fig. 4b) expression was not significantly different in skeletal muscle of OVX compared to sham-operated rats. In contrast, FNDC5 mRNA (Fig. 4c, *p ≤ 0.05) and protein (Fig. 4d, **p ≤ 0.01) content was significantly higher in skeletal muscle of OVX versus sham rats.

### Increased PGC-1α mRNA and reduced protein levels in skeletal muscle of testosterone treated orchiectomized rats

**Fig. 5:**
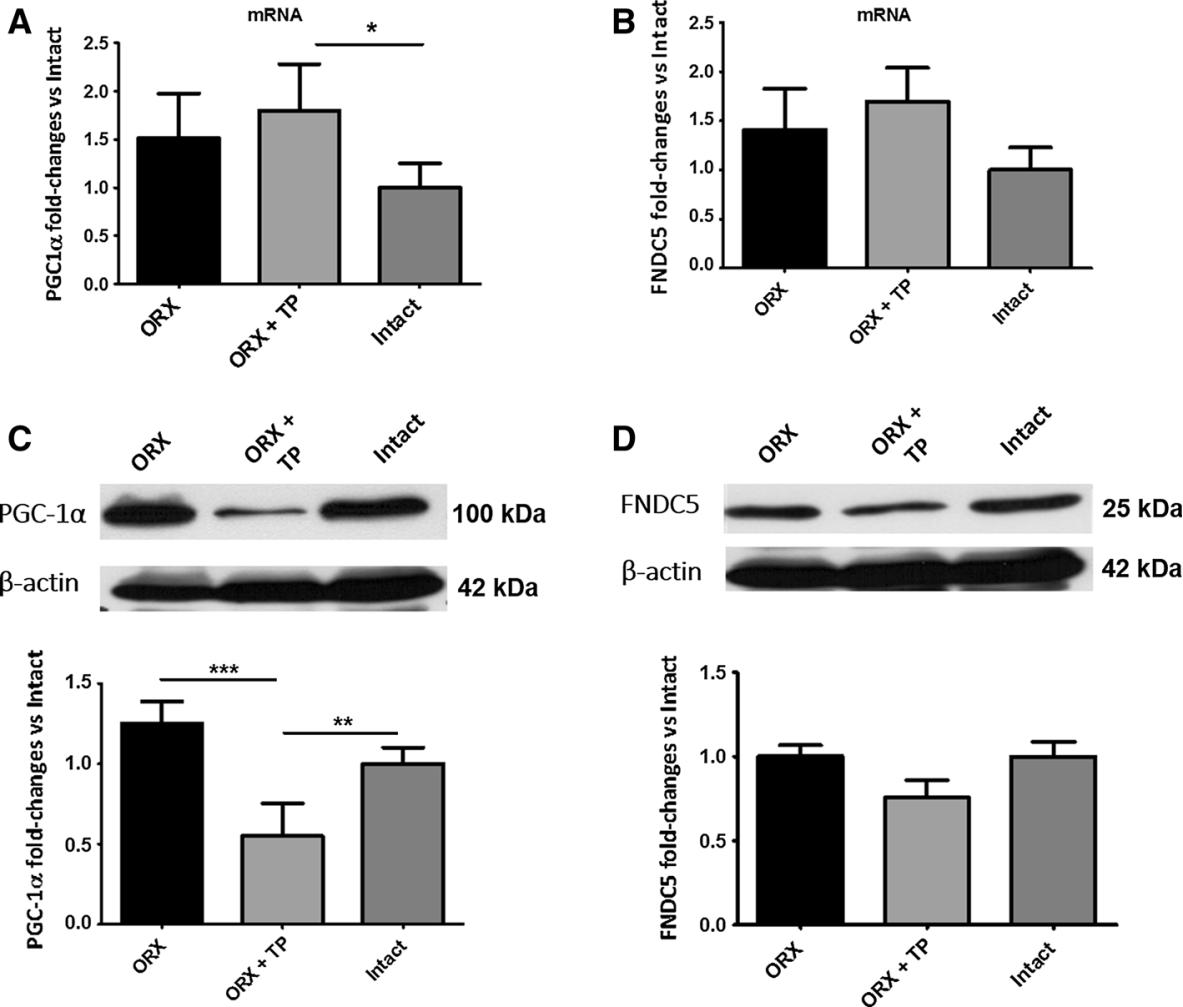
Effects of orchiectomy (ORX) on skeletal muscle PGC-1α and FNDC5 mRNA and protein content. Gene expression of PGC-1α (**a**) and FNDC5 (**b**). Western blot and densitometric analysis of PGC-1α (**c**) and FNDC5 (**d**) in skeletal muscle from ORX, ORX + TP and intact male rats, *n* = 6/group. **a** *p ≤ 0.05 ORX + TP versus intact, **c** ***p ≤ 0.001 ORX versus ORX + TP, **p ≤ 0.01 ORX + TP versus intact

PGC-1α mRNA content was significantly elevated in skeletal muscle of ORX + TP treated male rats compared to gonad intact male rats (Fig. 5a, *p ≤ 0.05). In contrast to elevated mRNA levels, protein expression was significantly reduced in ORX + TP versus ORX (Fig. 5c, ***p ≤ 0.001) and versus intact (**p ≤ 0.01). No significant differences were found between groups for FNDC5 mRNA (Fig. 5b) or protein content (Fig. 5d).

## Discussion

Recent studies suggest that there may be a sexual dimorphism in the regulation of irisin secretion. While our results and those of Scalzo et al. indicate no sex-specific differences for absolute resting irisin levels in healthy adults [31], other studies show that resting irisin was significantly higher in girls versus boys (12 ± 3 years old) and in healthy young (20 ± 0.1 years old) women versus men after adjusting for lean body mass [30]. Our data reveal however that there is a sexual dimorphism in the transient increase of irisin in response to acute, high-intensity endurance exercise: serum irisin concentrations were significantly higher immediately after intense endurance exercise in healthy, lean women versus men, suggesting that estrogens may play a role in the transient irisin increase. This is in line with findings by Scalzo et al. showing that 3 weeks of sprint interval training resulted in increased resting irisin levels in women but reduced levels in men 48 h after the last exercise bout [31]. Exercise intensity appears to be an important factor for irisin secretion, since no sex differences in irisin responses have been detected during low intensity acute endurance exercise [19]. Irisin concentrations increased 20 % in both men and women during moderate intensity endurance exercise and returned to baseline at the end of the exercise session [19]. In contrast to the acute elevations in serum irisin concentrations observed during and after intense endurance and resistance exercise [7, 20, 22, 23], resting irisin levels are either remained unchanged or reduced after chronic exercise training [7, 24]. This may be due to enhanced sensitivity of irisin action and/or a reduced need for irisin secretion after regular training due to the activation of other adaptive mechanisms stimulating numerous skeletal muscle signaling molecules responsible for mitochondrial biogenesis and enhanced glucose uptake, such as PGC-1α, TFAM, and GLUT-4 expression and translocation [25, 26, 33].

Interestingly, our results indicate that the sexually dimorphic response of irisin to acute exercise, observable in lean individuals, was blunted in severely obese (BMI >45) men and women. This outcome supports findings by Winn and colleagues, also demonstrating that irisin was not responsive to acute or chronic exercise in obese adults [34]. Moreover, in accordance with other studies [20, 35], total irisin concentrations at rest were higher in obese versus lean individuals, indicating that irisin is chronically elevated in obese people due to altered metabolic signaling associated with adiposity. However, even though exercise-induced changes may be small relative to the high baseline levels in obese individuals, even small irisin increases after exercise may still be biologically relevant for metabolic function. Furthermore, exercise has been shown to be able to increase irisin in subjects with metabolic syndrome [36].

### Gonadectomy, adiposity, and irisin

Huh et al. showed that a significant positive correlation exists between circulating irisin and estradiol [32] leading us to hypothesize that removal of estradiol by ovariectomy reduces irisin secretion. We used animal models mimicking the age-related sex hormone decline during menopause (ovariectomy, OVX) and andropause (orchiectomy, ORX) to study the effects of gonadectomy on irisin secretion. Declining female sex hormone in OVX rats levels mirror a pre-diabetic status [37] and are generally associated with increased adiposity and metabolic dysfunction [38]. Our results confirm that a lack of ovarian sex hormones as a result of OVX is associated with significant increases in body weight, of which increased visceral fat content is the major determinant. The amount of muscle mass relative to body weight was significantly reduced after OVX. In contrast, removal of male gonadal steroids in ORX rats had no significant effects on body weight, visceral fat content, or muscle mass. Contrary to our hypothesis, stating that particularly the removal of ovarian hormones would result in decreased irisin concentrations, significantly increased serum irisin levels were detected in OVX but not in ORX rats. This may be due to the increased adiposity and metabolic alterations associated with OVX and may be part of a complex compensatory mechanism in the early post-menopausal, pre-diabetic state to increase energy expenditure.

Cross-sectional studies have revealed that irisin concentrations were positively associated with biceps circumference, as a surrogate marker for muscle mass [32]. Rat skeletal muscle secretes approximately 60 % more irisin than visceral adipose tissue [16] and human *FNDC5* expression is 200-fold higher in skeletal muscle compared to adipose tissue [15, 16]. However, even though muscle mass was shown to be the strongest predictor of irisin levels [30, 32], irisin was not significantly reduced in sarcopenia patients [39]. This finding underlines the quantitative contribution of adipose tissue to irisin secretion, since sarcopenia is often associated with obesity known as sarcopenic obesity.

Our results did not confirm our hypothesis that removal of estradiol in OVX rats would reduce irisin secretion. In contrast, OVX was associated with significantly higher irisin level, suggesting that the adiposity-related molecular pathways associated with OVX stimulate chronic elevations in serum irisin. The type of adipose tissue may be relevant for irisin secretion patterns. Irisin was detected in the secretomes of both visceral and subcutaneous adipose tissue, and interestingly, subcutaneous adipose tissue secretes 40 % more FNDC5/irisin than visceral adipose tissue [16].

### Effects of gonadectomy on the regulation of the irisin signaling pathway: PGC-1α/FNDC5 expression

To elucidate the effects of gonadectomy on the signaling cascades leading to irisin concentrations, we investigated the effects of OVX/ORX on skeletal muscle PGC-1α and FNDC5 mRNA and protein levels. Our results indicate that while serum irisin and muscle FNDC5 are elevated at rest in ovariectomized female rats, PGC-1α expression was unaltered. Also, testosterone treatment in orchiectomized male rats significantly increased PGC-1α mRNA and reduced PGC-1α protein content without significantly altering FNDC5 mRNA or protein content. This implies that other, yet unidentified upstream molecules may be involved in activating FNDC5/irisin. Other studies using electrical stimulation of myotubes, exercise mimetics or exercise training in vivo have also shown PGC-1α upregulation does not necessarily lead to an activation of FNDC5 expression [6–9]. Recently, the extracellular signal-related kinase (ERK) signaling pathway has been suggested to control FNDC5 expression through a non-genomic pathway [10]. In addition, AMPK may be involved in the activation of FNDC5. FNDC5 expression in skeletal muscle was shown to be dramatically reduced in resting muscles of AMPK muscle-specific knockout mice compared to wild-type mice [12]. In response to muscle contractions, AMPK and FNDC5 activations were abolished even though PGC-1α was increased in wild-type and knockout mice, suggesting that AMPK is required for the regulation of FNDC5. Our results do not confirm a direct involvement of the PGC-1α/FNDC5/irisin pathway in response to gonadectomy, and more studies are needed to study the regulation of FNDC5/irisin in response to exercise or hormonal alterations.

## Summary

Taken together, we show that the transient increase in serum irisin after acute exercise is stronger in lean women compared to men, supporting findings of a positive association between estradiol and irisin [32]. Chronic elevations of irisin in obese versus lean individuals at rest may be mediated by altered metabolic signaling associated with adiposity; however, the exercise response was severely blunted in obese men and women. Exercise can be considered as a non-pharmacological treatment able to transiently increase serum irisin. Future studies should determine whether long-term exercise training combined with weight loss in obese individuals enhances the irisin response to exercise.

Lack of female sex hormones in OVX rats leads to adiposity and metabolic derailment, which is associated with increased circulatory irisin and skeletal muscle FNDC5 expression independent of PGC-1α expression. These findings point toward a compensatory mechanism in the early post-menopausal, pre-diabetic, and possibly ‘irisin-resistant’ state, where increased amounts of irisin are secreted in an attempt to increase energy expenditure by browning of WAT or other yet unidentified effects in skeletal muscle.
